# Depth-resolved partitioning of phytoplankton and bacterial communities in a seasonally stratified large lake

**DOI:** 10.1128/mra.01482-25

**Published:** 2026-04-27

**Authors:** Katelyn M. Brown, Mark J. Rozmarynowycz, Tijana Glavina Del Rio, Lars G. Rudstam, R. Michael McKay

**Affiliations:** 1 Great Lakes Institute for Environmental Research, University of Windsor 8637, Windsor, Ontario, Canada; 2 Department of Biological Sciences, Bowling Green State University 1888, Bowling Green, Ohio, USA; 3 Department of Energy, Joint Genome Institute 118576, Berkeley, California, USA; 4 Department of Natural Resources and the Environment, Cornell University 5922, Ithaca, New York, USA; Indiana University, Bloomington, Indiana, USA

**Keywords:** Lake Ontario, stratification, epilimnion, hypolimnion

## Abstract

Prokaryotic community composition was determined through V4 16S rRNA gene sequencing during early summer stratification in Lake Ontario. Despite a distinct thermocline, bacterial communities were stable, although differences were observed in chloroplast reads attributed to eukaryotic phototrophs. Chloroplast reads contributed up to 33.0% (station 63 epilimnion) of total reads.

## ANNOUNCEMENT

Warm monomictic lakes, like the Laurentian Great Lakes, alternate seasonally between isothermal mixing and thermal stratification (1). When mixed, phytoplankton occur throughout the water column, compared to stratified periods where chlorophyll *a* can be elevated below the thermocline, known as the deep chlorophyll layer (DCL) (2, 3). Since phytoplankton communities and nutrient availability differ between the surface and DCL, bacterial communities may also show patterns of partitioning. We applied 16S rRNA gene amplicon sequencing, metagenomics, and metatranscriptomics to investigate microbes in Lake Ontario’s surface waters and DCL.

**Fig 1:**
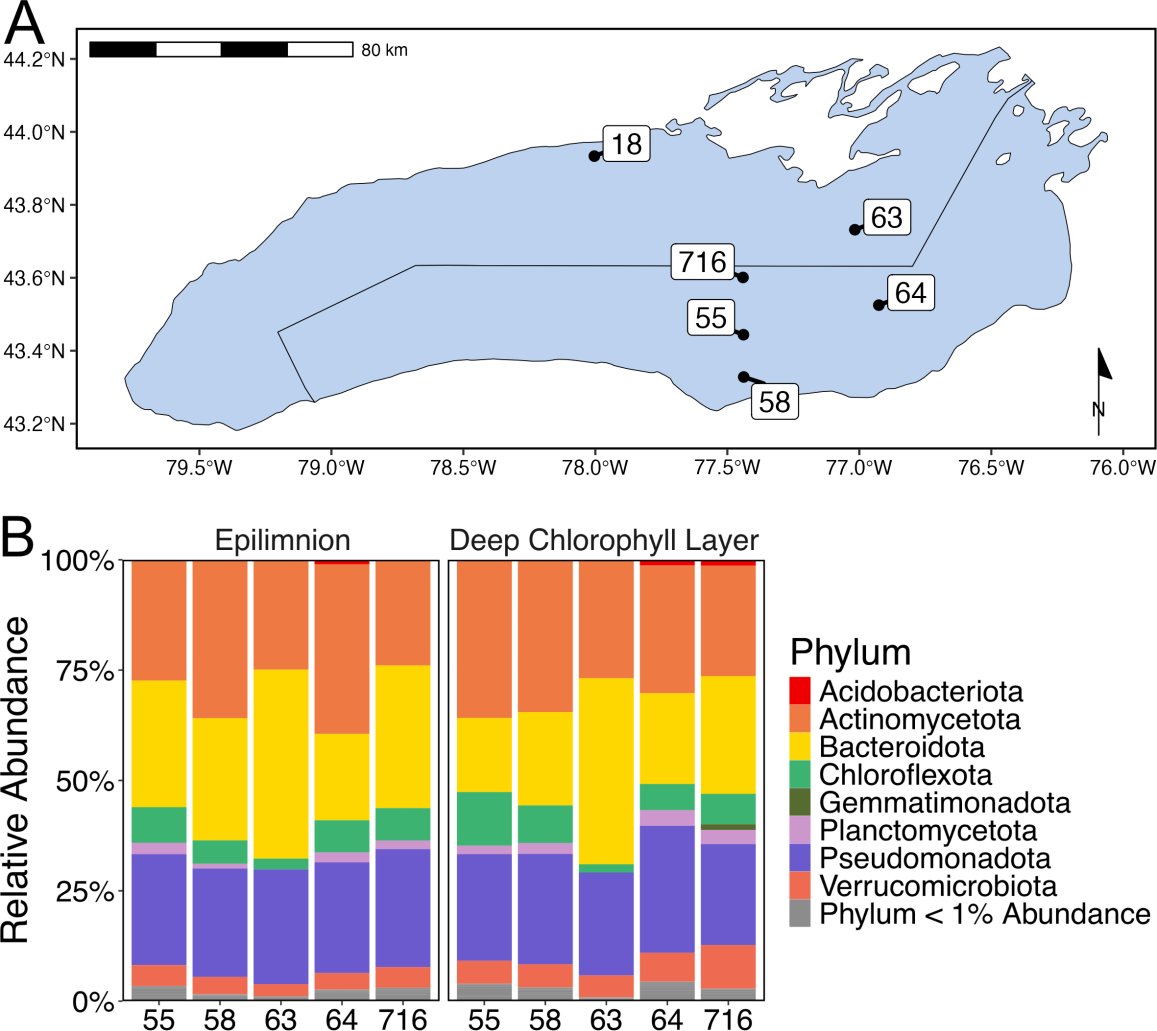
(**A**) Map of Lake Ontario with sampling stations overlaid. Five sites were targeted with amplicon sequencing, and one (station ON 18) was targeted with metagenomics and metatranscriptomics. (**B**) Relative abundance of prokaryotes at the phylum level for five sites with amplicon sequencing grouped by water column layer (epilimnion or deep chlorophyll layer). Taxa less than 1% abundant were aggregated at the phylum level.

Depth-integrated water samples were collected from the surface and DCL at five U.S. Environmental Protection Agency (EPA) stations onboard R/V *Lake Guardian* during the 2013 Lake Ontario Cooperative Science and Monitoring Initiative (Fig. 1A, Table 1) (4). Biomass was concentrated on 0.22 µm Sterivex filters (Sigma Aldrich, St. Louis, MO) and frozen in liquid nitrogen. DNA was extracted with the PowerWater Sterivex DNA Isolation Kit (MO BIO Laboratories, Inc., Carlsbad, CA). 16S rRNA gene V4 region sequencing was completed at the Joint Genome Institute (JGI; Berkeley, CA) on an Illumina MiSeq (2 × 250 bp; Illumina, San Diego, CA) (5, 6). Primers 515F (5′-CACGGTCGKCGGCGCCATT-3′) and a modified 806R (5, 6) were used with a HotMasterMix Amplification Kit (5PRIME, Montreal, QC). Sequences were processed with the dada2 v1.8 workflow (v1.36) (7), in R (v.4.5.1) (8),and RStudio (v.2025.05.1 + 513) (9). Chloroplast and mitochondrial reads were removed. Modifications to commands were filterAndTrim(trimLeft=c(21, 21), truncLen=c(240,240)), and mergers (minOverlap=20). Additional packages included phyloseq (v.1.52) (10), RColorBrewer (v.1.1-3) (11), ggh4x (v.0.3.1) (12), and tidyverse (v.2.0.0) (13). The map was created with sf (1.0-21) (14, 15), ggspatial (v.1.1.10) (16), ggplot2 (v.3.5.2) (17), and stringr (v1.5.1) (18).

**TABLE 1:** Location and summary of reads processing for sequenced samples^*a*^

Station ID—layer	Latitude	Longitude	Integrated sampling depths (m)	No. of raw reads	No. of reads after initial QC	No. of reads after removing chloroplast	No. of reads after removing chloroplast/ mitochondria	Library type	Accession no.
ON 18—epilimnion	43.933506	−78.003845	2, 5	141,179,563	N/A	N/A	N/A	Metagenomic	SRR3990180
ON 18—epilimnion	43.933506	−78.003845	2, 5	116,900,025	N/A	N/A	N/A	Metatranscriptomic	SRR5249012
ON 18—epilimnion	43.933506	−78.003845	2, 5	105,151,916	N/A	N/A	N/A	Metatranscriptomic	SRR5249013
ON 55—epilimnion	43.4439	−77.4389	2, 10	91,956	72,343	67,344	66,694	Amplicon	SRR35278446
ON 55—DCL	43.4439	−77.4389	15, 25, 30	96,079	81,060	72,876	72,228	Amplicon	SRR35278448
ON 58—epilimnion	43.328	−77.43791	2, 5	100,833	82,512	61,633	60,927	Amplicon	SRR1697709
ON 58—DCL	43.328	−77.43791	10, 20, 30	112,191	92,502	82,376	81,538	Amplicon	SRR35278441
ON 63—epilimnion	43.7317	−77.0169	2, 5	71,552	59,398	39,799	38,138	Amplicon	SRR35278432
ON 63—DCL	43.7317	−77.0169	15, 25	95,412	78,255	66,380	63,490	Amplicon	SRR1697717
ON 64—epilimnion	43.52495	−76.92603	2, 8	102,643	83,229	77,812	77,067	Amplicon	SRR35278439
ON 64—DCL	43.52495	−76.92603	15, 25, 35	103,162	81,323	68,279	67,740	Amplicon	SRR1697712
ON 716—epilimnion	43.60093	−77.4406	2, 5	116,220	98,186	84,614	83,762	Amplicon	SRR35278445
ON 716—DCL	43.60093	−77.4406	10, 20, 30	99,226	85,992	76,418	75,903	Amplicon	SRR35278447

^*a*^ Initial quality control (QC) consisted of filtering, trimming, dereplication, merging, and chimera removal. DCL, deep chlorophyll layer. N/A, not applicable.

Samples collected from the epilimnion from station ON 18 (May 2013) were filtered on a Sterivex and frozen in liquid nitrogen. DNA and RNA were extracted following references 19 and 20 with a DNeasy Tissue and RNeasy Mini Kit (Qiagen, Germantown, MD). DNA libraries were prepared with the KAPA-Illumina Library Creation Kit (Roche, Basel, Switzerland), and RNA libraries were rRNA depleted (Ribo-Zero rRNA Removal Kit; Illumina) and prepared with a TruSeq Stranded mRNA Kit (Illumina). Libraries were sequenced at JGI on an Illumina HiSeq 2500 (2 × 150 bp). Metagenomic assembly produced 2,170,353 contigs with an *N*_50_ of 492,865. Reads were error-corrected (bfc v.r181; kmer=21) (21) and assembled (SPAdes v.3.10.0-dev; --meta --only-assembler -k 21,33,55,77,99,127) (22), and filtered reads were mapped to the assembly (bwa v.0.7.15-r1142-dirty) (23). Contaminant contigs were removed (BBMap v.35.85). Default parameters were used unless otherwise noted. Metatranscriptomic reads were trimmed (ktrim=r, k=25, mink=12, tpe=t, tbo=t, qtrim=r, trimq=10, maq=10, maxns=3, minlen=50) and filtered (k=16) (BBDuk v.33.95). Contaminant rRNA reads and contigs were removed by mapping to the Silva database (v.119; BBMap v.33.95; fast=t, minid=0.90, local=t) and JGI contaminants database. Reads were concatenated, merged (BBMerge v.33.95), and normalized (BBNorm v.33.95) before assembly (Rnnotator v3.0.0) (24). The metatranscriptomes contained 206,160 and 244,117 contigs with N_50_s of 41,576 and 49,490. Bacterial phyla were consistent between the epilimnion and DCL (Fig. 1B), indicating a strong core microbiome in Lake Ontario.

## Data Availability

The amplicon sequencing project has been deposited in the NCBI Sequence Read Archive under the BioProject accession no. PRJNA255433. The station 18 metagenome and metatranscriptomes are found on GenBank under the BioProject accessions PRJNA329912 and PRJNA366186, respectively. The accession numbers are SRR3990180 (metagenome), SRR5249012 (metatranscriptome), and SRR5249013 (metatranscriptome). Assembled metagenomic and metatranscriptomic data can also be found in the JGI Integrated Microbial Genomes and Microbiomes (IMG) database under Taxon Object IDs 3300027836 and 3300003754, respectively. The raw Lake Ontario sequencing data can be found in the Joint Genome Institute Genomes OnLine Database (GOLD) under the GOLD Project IDs Gp0059905 (metagenome) and Gp0059906 (metatranscriptomes).

## EDITOR'S NOTE

**Editor's Note:** A potential conflict of interest was identified during the review of this paper, and the Editor in Chief performed an additional final review of the paper.
